# Halogen-directed drug design for Alzheimer’s disease: a combined density functional and molecular docking study

**DOI:** 10.1186/s40064-016-2996-5

**Published:** 2016-08-12

**Authors:** Adhip Rahman, Mohammad Tuhin Ali, Mohammad Mahfuz Ali Khan Shawan, Mohammed Golam Sarwar, Mohammad A. K. Khan, Mohammad A. Halim

**Affiliations:** 1 Division of Computer Aided Drug Design, Green Research Centre, BICCB, 38 Green Road West, Dhaka, 1205 Bangladesh; 2 Department of Biochemistry and Molecular Biology, University of Dhaka, Dhaka, 1000 Bangladesh; 3 Department of Biochemistry and Molecular Biology, Jahangirnagar University, Dhaka, 1342 Bangladesh; 4 Fakultät für Chemie und Biochemie, Organische Chemie I, Ruhr-Universität Bochum, Universitätsstrasse 150, 44801 Bochum, Germany; 5 Department of General Studies, Jubail University College, Jubail Industrial City, 31961 The Kingdom of Saudi Arabia; 6 Institut Lumière Matière, Université Lyon 1 – CNRS, Université de Lyon, 69622 Villeurbanne Cedex, France

**Keywords:** Alzheimer’s disease, Computer aided drug design, Density functional theory, Molecular docking, Nonbonding interactions, Halogenation

## Abstract

A series of halogen-directed donepezil drugs has been designed to inhibit acetyl cholinesterase (AChE). Density Functional theory (DFT) has been employed to optimize the chair as well as boat conformers of the parent drug and modified ligands at B3LYP/MidiX and B3LYP/6-311G + (d,p) level of theories. Charge distribution, dipole moment, enthalpy, free energy and molecular orbitals of these ligands are also investigated to understand how the halogen-directed modifications impact the ligand structure and govern the non-bonding interactions with the receptors. Molecular docking calculation has been performed to understand the similarities and differences between the binding modes of unmodified and halogenated chair-formed ligands. Molecular docking indicated donepezil and modified ligands had non-covalent interactions with hydrophobic gorges and anionic subsites of AChE. The –CF_3_-directed ligand possessed the most negative binding affinity. Non-covalent interactions within the ligand–receptor systems were found to be mostly hydrophobic and π- stacking type. F, Cl and –CF_3_ containing ligands emerge as effective and selective AChE inhibitors, which can strongly interact with the two active sites of AChE. In addition, we have also investigated selected pharmacokinetic parameters of the parent and modified ligands.

**Electronic supplementary material:**

The online version of this article (doi:10.1186/s40064-016-2996-5) contains supplementary material, which is available to authorized users.

## Background

Alzheimer’s disease (AD) is a neurodegenerative disorder that affects 5.4 million people and is the 6th leading cause of death in the United States alone. It is a form of dementia that worsens over time until a person can no longer have a conversation or respond to environmental stimuli. The major constituents of this disease are senile plaques and tangles that result in the death and damage of nerve cells through oxidative stress (Association 2012). Understanding the interaction between acetyl cholinesterase (AChE) and small molecules such as drugs which can inhibit this protein are also equally important to develop the therapeutic strategies against AD (McGleenon et al. 1999; Rees and Brimijoin 2003). Small drug molecules were successful to inhibit the acetyl cholinesterase (AChE) due to the presence of catalytic triad and aromatic gorge. These two most important binding sites are frequently targeted by AChE inhibitor drugs. The active catalytic triad, located near (~ 20 Å) the bottom of a deep and narrow gorge, consists of Ser200, His440 and Glu327 (Sussman et al. 1991). Aromatic gorge region which includes 14 aromatic amino acids such as Phe120, Phe288, Phe290, Phe330, Phe331, Trp84, Trp233, Trp279, Trp432, Tyr70, Tyr121, Tyr130, Tyr334, and Tyr442 and represents the 60 % of its total surface area (Xu et al. 2008). Similar to other protein, these aromatic amino acids are highly conserved. Among these aromatic amino acids, some process very distinctive functional activities. For instance, Trp84 and Phe330 are known as ‘‘anionic’’ subsite of the catalytic site (CAS) involves in choline recognition through cation‒pi interaction whereas Trp279 and Tyr70 contribute to the peripheral anionic site (PAS) (Gilson et al. 1994). Trp233, Phe288, Phe290 and Phe331 residues along with Gly119 also formed the acyl pocket involves in acetyl ester specificity (Harel et al. 1993, 1995).

Currently four drugs are marketed under different brands but are of limited or no benefits—three are acetyl cholinesterase inhibitors (rivastigmine, galantamine and donepezil) and the other (memantine) is an NMDA receptor antagonist (Birks and Harvey 2006; Pohanka 2011). These drugs normally have C-6 or C-5 ring based molecular structures with functional groups on the side chain. These are thought to prevent plaque formation and/or revert the mis-folding of the A-beta protein to its native 3-D structure. However, no drugs were found to significantly affect the symptoms or stop the progression of the disease in any clinical study. In recent years, significant research has been conducted to improve or discover new effective drugs using molecular modeling approach and laboratory extraction of natural products or modifying the currently available one (de Paula et al. 2007, 2009; Haviv et al. 2007; Nascimento et al. 2008; Sugimoto et al. 2002). For example, Farrokhnia and Nabipour reported acetyl cholinesterase inhibitors extracted from sea hare *Aplysiadactylomela* and studied their cholinergic actions by using molecular docking and density functional theory computations (Farrokhnia and Nabipour 2014). Camps et al. (2008) and Alonso et al. (2005) synthesized a series of donepezil-tacrine dimeric systems and tested their performance against Acetyl- and Butyrylcholinesterase.

Halogenation holds the promise of effective drug design by facilitating the drug molecules to cross biological barriers, filling small hydrophobic pockets present in protein targets, prolonging lifetime and easy adsorption. Being a strong electron-withdrawing group, halogens help in forming H‒bond and other non-covalent interactions (Lu et al. 2009, 2012; Politzer et al. 2007; Sarwar and Ajami 2013; Wilcken et al. 2012). Comparing with other halogenations, fluorination and carbon trifluoro-methylation have significant contributions to medicinal chemistry (Alonso et al. 2005; Gillis et al. 2015; Hagmann 2008; Zhou et al. 2009). Halogens stabilize the interactions of drug molecules with their protein target by promoting stronger bonding between functional groups through charge distribution. Further, some halogens such as I and Br contain regions with positively charge on them, which is responsible for its directional and stabilizing characteristics on the drug molecules (Kolář et al. 2013).

In this manuscript, we employ density functional theory to design some halogenated donepezil drugs. Earlier it was reported donepezil to show its antagonist activities against AChE while the piperidine ring being at chair conformation (Kryger et al. 1999). Here we have considered both the chair and the boat conformation of the piperidine ring prior to modifying the parent drug. Moreover, with the aid of molecular docking calculation, we report their interaction with different binding sites of AChE. These halogenated drugs show a considerable improvement in bonding with the target based on their structural features, which may help in developing an effective and low‒cost drug for Alzheimer’s disease.

## Computational methods

### Optimization of the ligands

All calculations were carried out using Gaussian 09 program package (Frisch et al. 2009). Initial three-dimensional geometry of chair forms of donepezil was retrieved from the bound crystal structure of 1EVE (Berman et al. 2002). The parent drug was modified with F, Cl, Br, I and –CF_3_ functional groups. These structures were fully optimized by density functional theory employing Becke’s exchange functional combining Lee, Yang, and Parr’s (LYP) correlation functional (Becke 1988; Lee et al. 1988). Midix basis set was employed for –Cl, –Br and –I substituted ligands, while 6-311G + (d,p) basis set was used for the parent drug and the –F and –CF_3_ modified derivatives (Easton et al. 1996). MidiX basis set is originally developed from the Huzinaga MidiX basis and applied to H, C–F, S–Cl, Br, and I atoms and can provide excellent geometries and charge balances with reasonable computational time and accuracy (Li et al. 1998). After optimization, subsequent vibrational frequency calculation has been performed to confirm that the stationary points correspond to minima on the Potential Energy Surface. Electronic energies, enthalpy, Gibbs free energies, and dipole moments and partial charge analysis of each compound were also investigated.

To predict the chemical reactivity descriptor of all ligands, molecular orbital calculations were performed at same level of theory. Hardness and softness of all drugs were also calculated from the energies of frontier HOMOs and LUMOs. Hardness (η) and softness (S) of the drugs calculated according to the following equation (Pearson 1986, 1995)

$$\begin{aligned} \eta & = \frac{\varepsilon LUMO - \varepsilon HOMO}{2} \\ S & = \frac{1}{\eta } \\ \end{aligned}$$

### Preparation of protein

The halogenated donepezil were subjected to molecular docking study against acetyl cholinesterase (AChE). Crystal structur of AChE were collected from the Protein Data Bank (PDB) database (PDB ID: 1EVE) (Berman et al. 2002). Prior to docking, water molecules were removed from the crystal structure followed by the addition of non-polar hydrogen atoms using AutoDock Tools (ADT) of MGL software packages (version 1.5.6). Later on, the fully optimized structures of the halogenated compounds were opened using ADT to add gasteiger charges and to set TORSDOF followed by the conversion of all rotatable bonds into non-rotatable. Next, we saved both the protein and ligand structures in .PDBQT format as it is the only one supported file format that required by AutoDock Vina (version 1.1.2, May 11, 2011) for docking analysis (Trott and Olson 2010).

### Binding site and docking

The active binding pocket of AChE is predicted by CASTp—having the highest pocket area and volume are 763.5 Å^2^ and 1716.4 Å^3^, respectively (Dundas et al. 2006). The binding pocket and the amino acid residues are presented in Additional file 1: Figure S1 (supporting information). These residues have been identified to contribute to the structural and functional properties of the protein by catalytic tirade and most of the aromatic gorge. The binding site residues predicted by CastP for AChE were used for grid generation.

While docking ligands against AChE, center grid box was set at 65.2551, 63.0417 and 59.0772 Å. Autodock Vina docking protocol was employed to conduct the docking study. The docked pose of lowest binding free energy conformer with the respective protein was analyzed using PyMOL Molecular Graphics System (version 1.7.4) (DeLano 2002), Accelrys Discovery Studio 4.1 (“Accelrys Software Inc., Discovery Studio Modeling Environment, Release 4.0, San Diego: Accelrys Software Inc.,” 2013).

### Pharmacokinetic parameters study

We have utilized AdmetSAR online database to evaluate the pharmacokinetic parameters related to drug absorption, metabolism and toxicity for the parent drug and its modifiers (Cheng et al. 2012). Prior to that, SDF (Structure Data File) and SMILES (simplified molecular- input line-entry system) strings were utilized throughout the generation process.

## Result and discussion

**Fig. 1:**
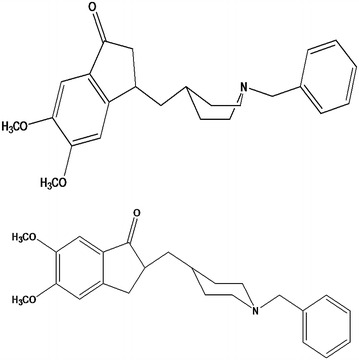
Chair and boat conformations of Donepezil

**Fig. 2:**
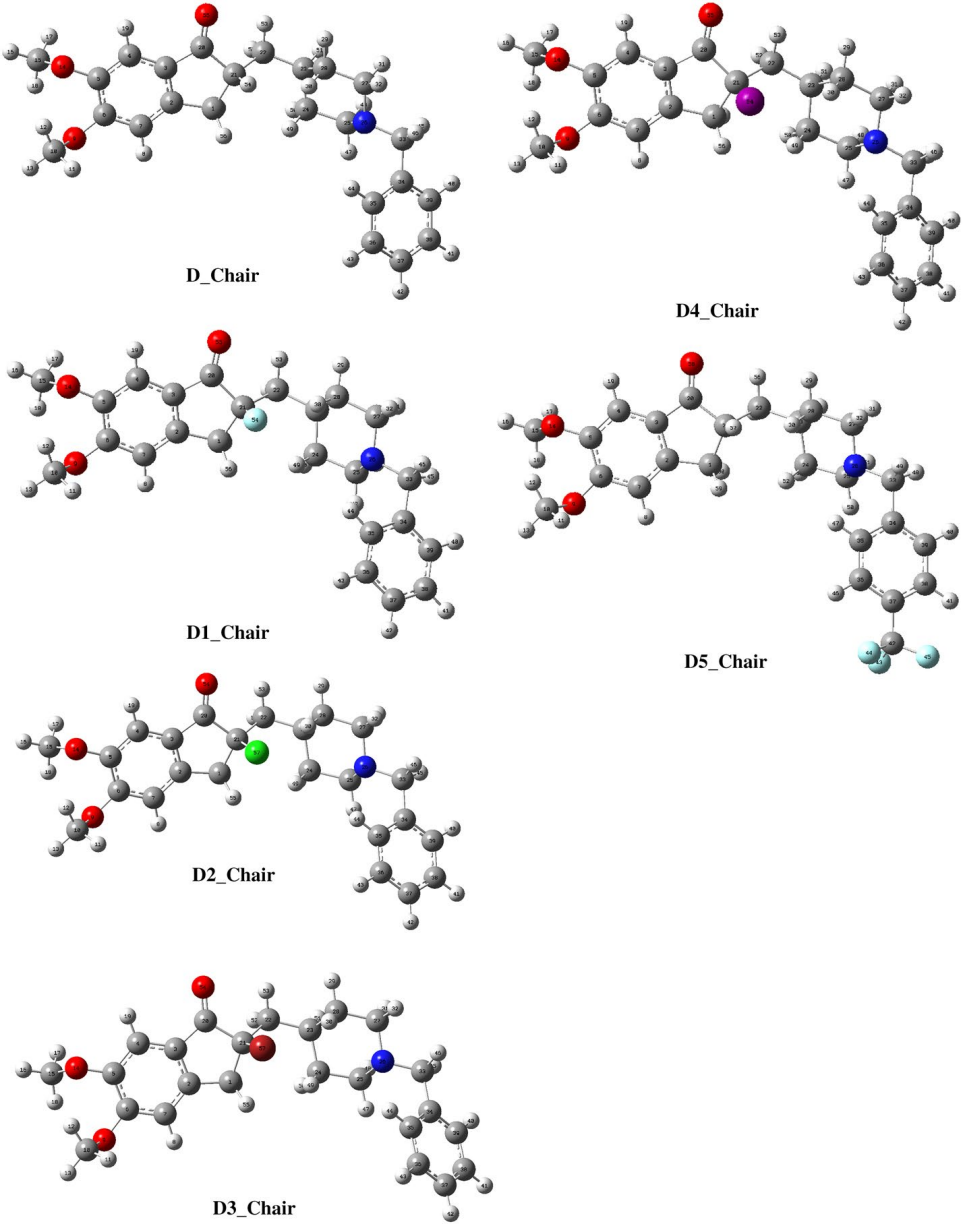
Optimized structure of Donepezil (D) Chair form and its halogenated derivatives (D1, D2, D3, D4 and D5) calculated at B3LYP/MidiX level of theory

Figure 1 shows two dimensional structures of donepezil in both chair and boat forms. The optimized structure of donepezil (D) and its halogenated derivatives (D1-D5) at chair form are presented in Fig. 2. The stoichiometry, electronic energy, enthalpy, Gibbs free energy and dipole moment of the ligands are reported in Table 1. The HOMO and LUMO energy values, the energy gap, and softness of all ligands are presented in Table 2. The binding affinity and important non-covalent interactions of all ligand–receptor complexes are summarized in Tables 3 and 4 respectively.

**Table 1:** The stoichiometry, electronic energy, enthalpy, Gibbs free energy in Hartree and dipole moment (Debye) of donepezil chair form and its derivatives

Name	Stoichiometry	Electronic energy	Enthalpy	Gibbs free energy	Dipole moment
D_Chair	C_24_H_29_NO_3_	−1204.887	−1204.887	−1204.970	2.58
D1_Chair	C_24_H_28_FNO_3_	−1303.572	−1303.571	−1303.656	3.82
D2_Chair	C_24_H_28_ClNO_3_	−1662.323	−1662.322	−1662.409	4.31
D3_Chair	C_24_H_28_BrNO_3_	−3766.304	−3766.303	−3766.390	3.78
D4_Chair	C_24_H_28_INO_3_	−8094.427	−8094.427	−8094.515	3.77
D5_Chair	C_25_H_28_F_3_NO_3_	−1540.040	−1540.038	−1540.132	1.64

**Table 2:** Energy (atomic unit) gaps of HOMOs, LUMO, Gap, Hardness and Softness of all drugs

Molecules	*ε*_*HOMO*_	*ε*_*LUMO*_	*Gap*	*S* (Softness)
D_Chair	−0.21374	−0.04412	0.16962	11.79106
D1_Chair	−0.20857	−0.05763	0.15094	13.26787
D2_Chair	−0.21086	−0.06102	0.14984	13.34757
D3_Chair	−0.21101	−0.06008	0.15093	13.25118
D4_Chair	−0.21245	−0.06621	0.14624	13.67614
D5_Chair	−0.22074	−0.04579	0.17495	11.43183

**Table 3:** Free energy of binding values (Kcalmol^−1^) for ligand – AChE (at chair and boat form) systems obtained from flexible docking

Systems	Free energy of binding	
	Chair	Boat
D-AchE	−11.1	−11.5
D1-AchE	−11.7	−12.6
D2-AchE	−11.5	−12.0
D3-AchE	−11.2	−11.2
D4-AchE	−10.2	−11.0
D5-AchE	−12.0	−12.5

**Table 4:** Selected non-covalent interactions among chair ligands D–D5 and AChE obtained via flexible docking

Systems	Contacts	Bond distances (Å)	Systems	Contacts	Bond distances (Å)
D-AchE	C–H···O (Tyr70)	2.66	D3-TYMS	C–H···O (Asp72)	2.94
	pi···pi (Trp84)	3.85		pi···pi (Trp84)	3.90, 4.01
	pi···pi (Trp279)	4.14		pi···pi (Trp279)	4.41, 5.61
	O···H–N (Phe288)	2.84		C–H···O (Ser286)	2.81
	pi···pi (Phe330)	4.03		C–H···pi (Phe330)	2.33
	pi···pi (Phe331)	5.30		pi···pi (Phe331)	5.43
	pi···pi (Tyr334)	4.65		Alkyl···pi (Tyr334)	4.54
D1-AchE	C–H···O (Asp72)	2.90	D4-AChE	pi···pi (Trp84)	3.75
	pi···pi (Trp84)	3.87, 3.98		Alkyl···pi (Phe330)	4.92
	pi···pi (Trp279)	4.41, 5.66		Alkyl···pi (Phe331)	5.20
	C–H···O (Ser286)	2.90		Alkyl···pi (Phe334)	5.41
	C–H···pi (Phe330)	2.40			
	Alkyl···pi (Phe331)	5.41			
	Alkyl···pi (Tyr334)	4.53			
D2- AchE	C–H···O (Asp72)	2.70	D5-AChE	C–H···O (Asp72)	2.74
	pi···pi (Trp84)	3.89, 4.00		pi···pi (Trp84)	3.93, 4.17
	pi···pi (Trp279)	4.35, 5.60		F···H–C, F···C (Gly117)	2.63, 3.17
	C–H···O (Ser286)	3.00		F···H–O (Tyr130)	2.93
	O···H–N (Phe288)	2.71		F···O (Glu199)	2.86, 2.91
	C–H···pi (Phe330)	2.40		pi···pi (Trp279)	4.10
	pi···pi (Phe331)	5.29		C–H···O (Ser286)	2.74
	pi···pi (Phe334)	4.49		O–H···N (Phe288)	2.42
				pi···pi (Phe330)	4.23
				C–H···pi (Phe331)	2.92
				pi···pi (Tyr334)	4.44

Brackets indicate the amino acid residues that are in contact with the ligands

*Asp* asparatic acid, *Gly* glycine, *Glu* glutamine, *Phe* phenylalanine, *Ser* serine, *Trp* tryptophan, *Tyr* tyrosine

### Binding affinity of Donepezil (D) and modified drugs (D1–D5) in chair form against AChE

**Fig. 3:**
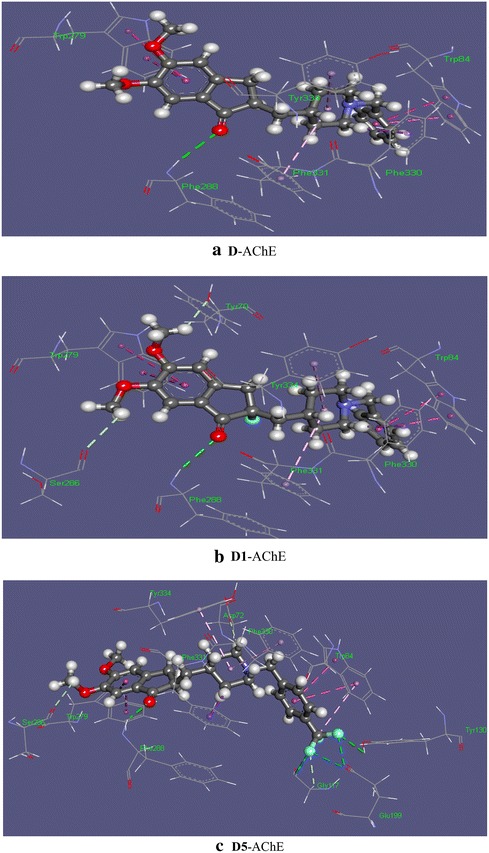
Non-covalent interaction maps of **a** D (Chair)-AChE, **b** D1 (Chair)-AChE and **c** D5 (Chair)-AChE complexes

Halogen-directed modifications on donepezil significantly influence the structural properties of the ligands in terms of energy, partial charge distribution and dipole moment. In D1, introducing a fluorine atom at 54 positions replacing the H atom changes the free energy to −1303.57 Hartree from −1204.88 Hartree. In D1, the dipole moment increases by 1.25 D compared to donepezil. This may play a role in determining the activity of ligands as elevated dipole moment has been considered a good indicator to promote non‒bonded interactions in drug-receptor complexes (Lien et al. 1982). F atom interchanges the partial charge on C21 to +0.215 a.u. from −0.326 a.u. with a partial charge of −0.308 a.u. on F. Inclusion of F kept a role to increasing the softness of D1 (13.27) compared to that of the parent ligand D (11.79). The H54–C21 bond distance (1.09 Å) increases when H is replaced by F to 1.41 Å. Gibbs free energy of the parent drug went towards more negative value when H54 had been replaced by F in D1. Binding affinity of D and D1 against AChE was computed as −11.1 and −11.7 kcalmol^−1^ respectively, which demonstrated the fact that inclusion of a high electronegative group affects the overall ligand–receptor interaction scenario. Farrokhnia and Nabipour (2014) docked donepezil with AChE and found the binding energy value to be −11.2 kcalmol^−1^ which is in good agreement with our computed value. In D-AChE complex, Phe330 in AChE has previously been described by Kryger et al. as a “swinging gate” and its tendency to alter conformation has been anticipated to having an important role in the activity of the ligand. They also indicated of the presence a pi‒stacking interaction involving the phenyl ring at Phe330 (Kryger et al. 1999). In the present work, the pi‒pi interaction at Phe330 has been observed with the bond distance being 4.03 Å (Fig. 3). This was however in contrast with the experimental observation by Kryger et al. where the interaction had been at the piperidine nitrogen. A good number of pi‒pi interactions were observed that show the bond distance varying between 3.80 Å to as far as 5.00 Å. Some recent researches, however, indicated that ideal bond distance in pi‒pi interactions are within the range 3.30–4.00 Å (Janiak 2000; Martinez and Iverson 2012; Avasthi et al. 2014). The optimized geometry of donepezil was not found to show any contact with the so-called catalytic triad consisting three amino acid residues Ser200, His440 and Glu327 as noted by Kryger et al. Instead, parent drug D and the modified ligands mostly showed good contacts with the aromatic amino acid gorge involving mainly pi‒stacking interactions. For example, parent drug D was found to generate stacking contacts with the delocalized electrons at indole fragment of Trp84 and Trp279, the aromatic ring of Phe331 and Tyr334. Earlier Trp84 was reckoned as one of the important residues of the active site of gorge. Replacing Trp84 by alanine significantly reduced the catalytic activity of human AChE (Ordentlichs et al. 1993). In the same work, Kryger et al. predicted of a finger‒shaped void, defined by Phe288, Phe290, Phe331 and Trp233, at the acyl-binding pocket. X-ray crystallography and photoaffinity labeling study exhibited that Trp84 and Phe330, known as “anionic” subsite of the active site of AChE, plays a pivotal role in ligand binding. Moreover, residue Trp279, part of peripheral anionic site located in the top of the gorge, also stabilizes the ligand (Farrokhnia and Nabipour 2014). The only interaction site was observed at the Phe288 residue with the O^…^H‒N bond distance being 2.84 Å. F substituted ligand D1, on the other hand, was found to generate multiple hydrogen bonding with Tyr70, Ser286 and Phe288 as shown in Fig. 3b. Both Tyr70 and Ser286 were involved in forming C‒H^…^O interaction with the C atoms of the two –OCH_3_ groups, however, that was not observed at the same sites of parent drug D. The bond lengths of the two contacts, 2.70 and 2.90 Å respectively, showed that the C‒H^…^O interactions were moderately strong. The C‒H^…^pi stacking contact involving the piperidine ring of D1 and Phe330 became slightly more intact, as the bond length reduced to 2.40 Å compared to that in the D-AChE complex. In terms of bond-lengths, T shaped pi‒pi stacking contacts at Phe331 and Tyr334 did not show any significant variation from that of the D-AChE system. Here it is worth noting that Kryger et al. predicted of a finger‒shaped void, defined by Phe288, Phe290, Phe331 and Trp233, at the acyl-binding pocket and our computation hinted that the ligands actually approach the void by generating non‒covalent interactions with some of the amino acids surrounding the pocket. Like the parent drug, D1 showed stacking interactions with the indole fragment of Trp84. It is interesting to note that F did not take part in any contact with the amino acid residues but, as one could see, its inclusion influenced the spatial arrangement of the drug, binding affinities, introduction to new contact sites and nature of non‒bonding contacts.

The binding affinities of the Cl, Br and I substituted ligands (D2-D4) were lower compared to that of D1, which could be attributed to the comparatively lesser electronegativity and larger van der Walls Radii of Cl, Br and I than that of F. In terms of the electronic and thermodynamic properties of the ligands, as listed in Table 1, non-bonding interaction maps for these three molecules were given in Additional file 1: Figure S2. Ligand D2 had more favorability albeit it’s binding affinity to AChE being slight lower than that of D1. Contacts with the amino acid residues remained almost same to the former two ligands except an additional C‒H^…^O interaction at Asp72. The C–H^…^pi bond‒length at Phe330 became 2.35 Å, which further reduced when Cl was substituted by Br in ligand D3. Contacts at Trp279, Phe331 and Tyr334 did not see any abrupt change in both D2 and D3. Potential energy surface of the iodine‒substituted ligand D4 showed the electron density be slightly positive over I. Moreover, D4 possessed lower dipole‒moment and was less soft than the other molecules (Table 2). This might have determined its low binding affinity, which was −10.2 Kcalmol^−1^. The value, for instance, was about 1.5 Kcalmol^−1^ more positive than its fluorinated counterpart D1. D4 formed a pi‒pi stacking interaction (4.92 Å) with Phe330 instead of forming a C‒H^…^pi interaction. The number of total interactions that D4 formed with AChE was significantly less than that of the previous ligands. D4 also did not approach any amino acid at the void of the acyl-binding pocket. Moreover, no hydrogen-bonded residual site involving D4 was found to exist.

**Fig. 4:**
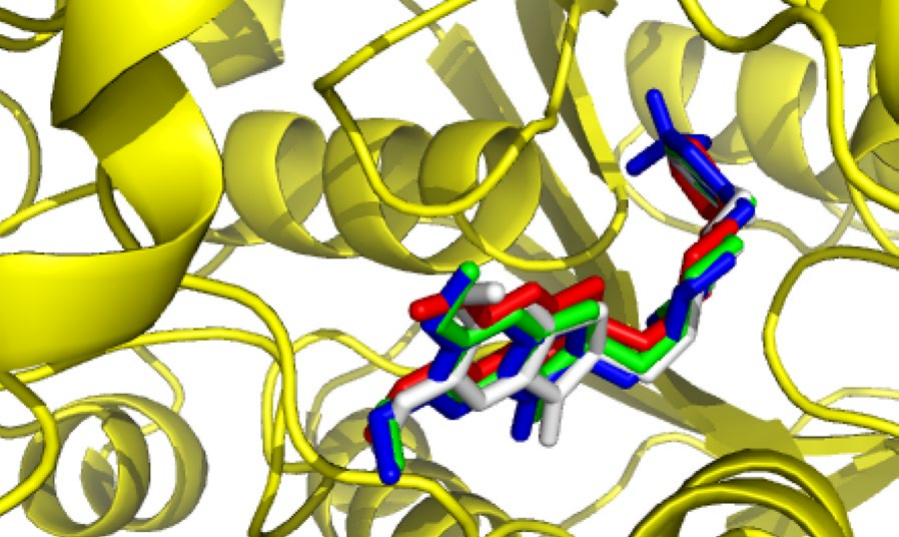
Superposition of the chair conformers of D (*green*), D1 (*white*) and D5 (*blue*) on the E2020 (*red*)/AChE crystal structure resolved at 2.5 Å

In ligand D5, trifluoromethyl (–CF_3_) group was incorporated to C37. –CF_3_ has been introduced to different organic molecules for applications in agrochemical, dyes and pigments, pharmaceuticals, polymers, and material chemistry (Furuya et al. 2011; Ji et al. 2011; Roy et al. 2011). Due to strong electronegativity and lipophilic nature, the application of trifluoromethyl group in drug design can promote selective functionality related to physiochemical, biological and pharmacological properties (Lishchynskyi et al. 2013). Incorporating –CF_3_ group in C37 position significantly influenced the structure of donepezil. –CF_3_ group significantly increased the dipole moment (5.857 Debye) and enhanced the polar nature of D5. In the D5-AChE docked structure, non-bonded interactions were diverse. The interaction with the swinging Phe330 was a pi‒pi stacking with a bond-distance 4.92 Å. D5 also formed non‒bonded interactions with Asp72, Trp279, Ser286, Phe288, Phe331 and Tyr334 which means that it had covered most of the possible voids and pockets where the ligand could be situated in. The nature of those interactions was mostly hydrophobic. Details about the types of interactions and bond‒distances are given in Table 4 and Fig. 3c. The most notable fact here is, however, that the three F atoms of the –CF_3_ group in benzyl ring interacted with amino acids to form strong halogen interactions. F atoms held Gly117, Tyr130 and Glu199 at multiple binding sites resulting in the presence of O^…^F, C‒H^…^F and O‒H^…^F interactions. The residues were near the part known as “oxyanion hole” formed by the peptide NH moieties of Gly118, Gly119 and Ala201 and having an essential role in catalysis (Tormos et al. 2010). These non‒covalent interactions were from moderately strong to fairly strong with the bond distance being ranging from 2.65 to 3.24 Å. The abundance of non‒bonding contact sites might explain the fact that binding affinity for D5-AChE had come highest among the ligand–receptor systems considered for the present work (−12.0 KCalmol^−1^). Figure 4 shows that D, D1 and D5 superimpose well on the experimental crystal structure of E2020/AChE resolved by Kryger et al.

### Interaction and binding affinity of the ligands in boat form (D^ʹ^–D^ʹ^5) against AChE

The boat forms of donepezil and the modified derivatives were due to the change of conformations from chair to boat at the piperidine ring. Additional file 1: Table S1 describes the thermodynamic and electronic properties of the boat conformers D^ʹ^–D^ʹ^5. Free energies of the boat molecules were slightly more negative compared to the chair counterparts. The dipole moments and softness values for the boat conformers had larger values than the corresponding chair conformers. For instance, dipole moment and softness values of the boat conformer of donepezil were 3.57 and 12.16 D respectively which are almost 1.0 and 0.40 D units larger than that of chair donepezil. The pattern of the changes of the electrical and thermodynamic parameters for the D^ʹ^–D^ʹ^5 were identical to the D–D5 counterparts, which was demonstrated by the increasing dipole moment and softness up to D^ʹ^4 and a decrease for –CF_3_ modified D^ʹ^5. The most notable part regarding the boat conformers was that binding affinity for each of the molecules was 0.2‒1.0 Kcalmol^−1^ more negative compared to the counterpart chair conformers. The most favorable boat ligands according to molecular docking were fluorinated and –CF_3_ modified D^ʹ^1 and D^ʹ^5 forms with binding energies being −12.6 and −12.5 Kcalmol^−1^ respectively. Comparison among the energy values has been shown in Table 3. Such binding affinity, however, was in less concordance when correlated with the non-covalent interactions found for (D^ʹ^–D^ʹ^5)-AChE complexes. According to the binding site analysis, Donepezil at the boat conformer was found to form a few hydrophobic contacts and C‒H^…^O bonding with Trp279, Arg289 and His440. The latter two amino acids were not involved at the contact sites involving any of the chair conformers and His440 is one of the three amino acids of the catalytic-triad. Analysis of D^ʹ^1, on the other hand, saw the formation of pi‒stacking interactions with Trp84, Tyr334 and Gly335. The C‒H^…^pi bond distance at Tyr334 was found to be 2.70 Å. Ligand D^ʹ^5, on the other hand, showed stacking interactions with Trp84, Phe330, Phe331 and Tyr334; moreover, the F atoms formed a number of H‒bonds with Trp84 and Gly118. Details about the nature of the contact sites for the boat conformers D^ʹ^, D^ʹ^1 and D^ʹ^5 against AChE are given in Additional file 1: Figure S3. D^ʹ^2–D^ʹ^4 showed some identical binding sites to that of their chair counterparts- stacking interactions with Trp279, Phe330 and Tyr334 for example. In addition to that, some hydrophobic contacts and H‒bonding at Trp8, Tyr121 and Asp285 were observed.

### Pharmacokinetic parameters of the chair conformers

Our ADME (absorption, distribution, metabolism, and excretion) evaluation shows that all the chair forms of the drugs are non‒carcinogenic having a class III acute oral toxicity shown in Table 5. LD50 values for the molecules are above 2.8378 mol/Kg; trifluoromethyl substituted D5 has the highest LD50 value within the class (3.0139 mol/Kg) indicating D5 to be the best modified ligand for in vivo studies. As the human intestinal absorption values were found positive for all the ligands and all of the ligands are P‒glycoprotein inhibitor, it can inferred that the drugs will act positive in terms of bioavailability, drug metabolism and intestinal absorption (Broccatelli et al. 2011; Shen et al. 2010). This can be further reinforced by the fact that donepezil and the modified derivatives shows positivity towards blood brain barrier (BBB) predicting the fact that all of them are supposed to go through BBB. One disadvantage found for donepezil is that it shows strong hERG inhibitory properties, which is responsible for adverse drug–drug interactions and cardiac side-effects (Hundae et al. 2014). The modified molecules are, however, found to be weak hERG inhibitor.

Inhibition constant for the drugs have been calculated using concept of equilibrium between enzyme and its inhibitor

$${\text{E}} + {\text{I}} \leftrightarrow {\text{EI}}$$

(E = Enzyme and I = Inhibitor; the reference concentrations for all the entities have been considered 1 molL^−1^ for the calculations) and the relationship-

$$\ln \,K_{b} = - \ln \,K_{i}$$

where ln K_b_ = −ΔG/RT and ΔG = free energy of binding and are presented in the last row of Table 5.

**Table 5:** Selected pharmacokinetic parameters of Donepezil (Chair Form) and its derivatives

Parameters	Donepezil	D1	D2	D3	D4	D5
Blood Brain Barrier	+ (0.9953)	+ (0.9931)	+ (0.9921)	+ (0.9911)	+ (0.9887)	+ (0.9941)
Human Intestinal Absorption	+ (0.9566)	+ (0.9962)	+ (0.9963)	+ (0.9946)	+ (0.9802)	+ (1.0000)
P-glycoprotein Inhibitor	Inhibitor (0.7641)	Inhibitor (0.8388)	Inhibitor (0.7572)	Inhibitor (0.8138)	Inhibitor (0.8185)	Inhibitor (0.7949)
Human Ether-a-go–go-Related (hERG) Gene Inhibition	Strong-inhibitor (0.5386)	Weak-inhibitor (0.6930)	Weak-inhibitor (0.5693)	Weak-inhibitor (0.6698)	Weak-inhibitor (0.6194)	Weak-inhibitor (0.6544)
Acute Oral Toxicity	III	III	III	III	III	III
Rat Acute Toxicity, LD50 (mol/Kg)	3.0123	2.8808	2.8378	2.8435	2.8480	3.0139
Ki (at 298 K, nM)	7.442	2.705	3.789	6.285	33.955	1.631

Probability values related to each of the parameters are given in the parenthesis

## Conclusion

Our study demonstrated the binding interactions of halogenated donepezil ligands in chair form with AChE. Overall, halogenation increased the dipole moment of the modified ligands thereby enhancing their polar nature. Moreover, halogenation made the modified ligands thermodynamically more stable as evident from enthalpy and Gibbs free energies. The HOMO‒LUMO energy gaps of these modified ligands were reasonably lower than donepezil, which indicated that these compounds are more chemically reactive. The –CF_3_ modified ligand D5, however, showed some degree of anomaly from the pattern observed; however, its binding affinity to AChE was mostly favorable. The study also indicated that Br and I directed modifications did not provide performances as the F and –CF_3_ directed modifications did. Non‒covalent interactions such as pi‒pi stacked, pi‒pi T‒shaped, amide‒pi stacked and pi‒alkyl alongside C‒H^…^O and N‒H^…^O interactions were the main driving force of the enhanced performance of D1 and D5. The C‒H^…^pi interaction at Phe330 residue for D‒D3 showed strong interactions with the bond distances being ranged between 2.35‒2.47 Å. The boat conformers showed increased binding affinity than the chair conformers despite the fact that binding sites for the boat ligands were not entirely similar to the chair counterparts.

## Additional file

10.1186/s40064-016-2996-5 depict binding pockets and non-bonding interactions. **Table S1** shows the electronic and thermodynamics properties of the boat conformer of donepezil and **Table S2** presents the rigid docking binding energy for the chair conformer of donepezil.
