# Supplementary material for: Halogen-directed drug design for Alzheimer’s disease: a combined density functional and molecular docking study
Source: Springerplus. 2016 Aug 12;5(1):1346. doi: 10.1186/s40064-016-2996-5 (PMC4987752; doi:10.1186/s40064-016-2996-5)
Supplement: Supplementary file 1 — 10.1186/s40064-016-2996-5 depict binding pockets and non-bonding interactions. Table S1 shows the electronic and thermodynamics properties of the boat conformer of donepezil and Table S2 presents the rigid docking binding energy for the chair conformer of donepezil. [file 40064_2016_2996_MOESM1_ESM.docx]

Supporting Information

**Halogen-directed Drug Design for Alzheimer’s disease: A combined Density Functional and Molecular Docking Study**

Adhip Rahman^1^_,_Mohammad Tuhin Ali^1,2ǂ^,Mohammad Mahfuz Ali Khan Shawan^1,3^, Mohammed Golam Sarwar^4^ , Mohammad A K Khan^5^ Mohammad A. Halim^1,6*^

^1^Division of Computer Aided Drug Design, BICCB, Green Research Centre, 38 Green Road West, Dhaka-1205, Bangladesh

^2^Department of Biochemistry and Molecular Biology, University of Dhaka, Dhaka-1000, Bangladesh

^3^Department of Biochemistry and Molecular Biology, Jahangirnagar University, Dhaka-1342, Bangladesh

^4^Fakultät für Chemie und Biochemie, Organische Chemie I, Ruhr-Universität Bochum, Universitätsstrasse 150, 44801 Bochum, Germany

^5^Jubail University College, Department of General Studies, Jubail Industrial City 31961, The Kingdom of Saudi Arabia

^6^Present Address: Institut Lumière Matière, Université Lyon 1 – CNRS, Université de Lyon, 69622, Villeurbanne Cedex, France

*Corresponding authors. E-mail: [mohammad-abdul.halim@univ-lyon1.fr](mailto:mohammad-abdul.halim@univ-lyon1.fr)(MAH)


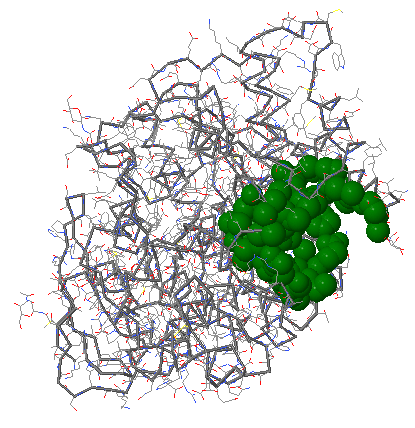


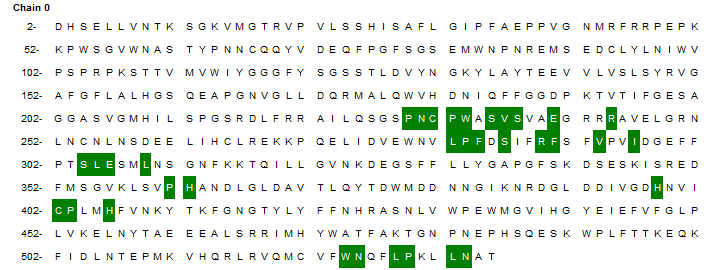


**Figure S1:** The binding pocket of AChE

**
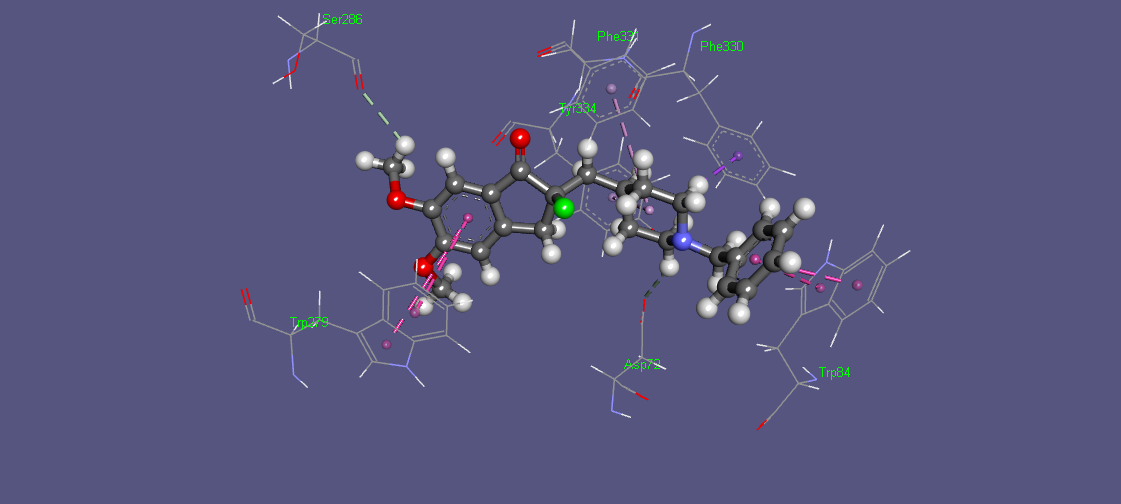
**

(a) **D2**- AChE

**
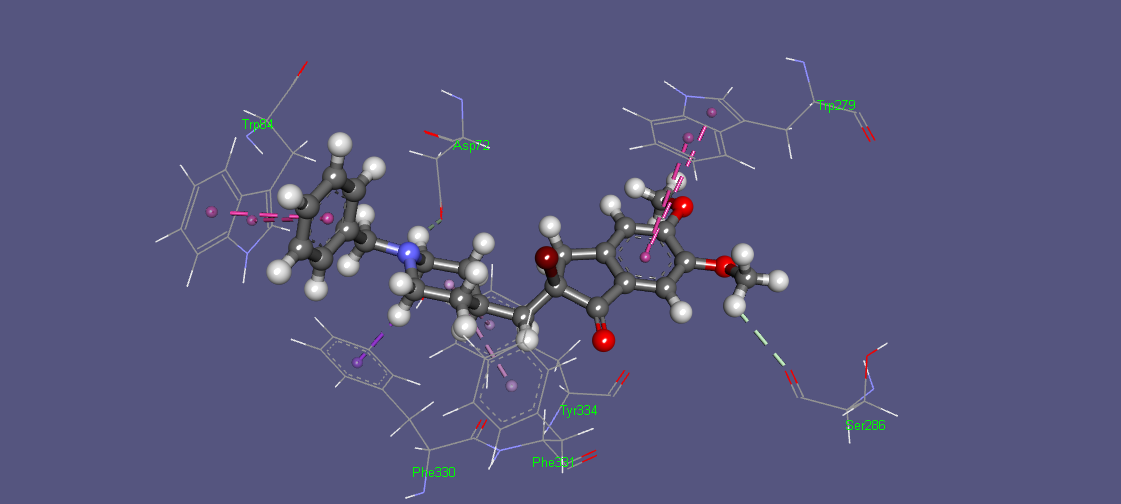
**

(b) **D3**- AChE

**
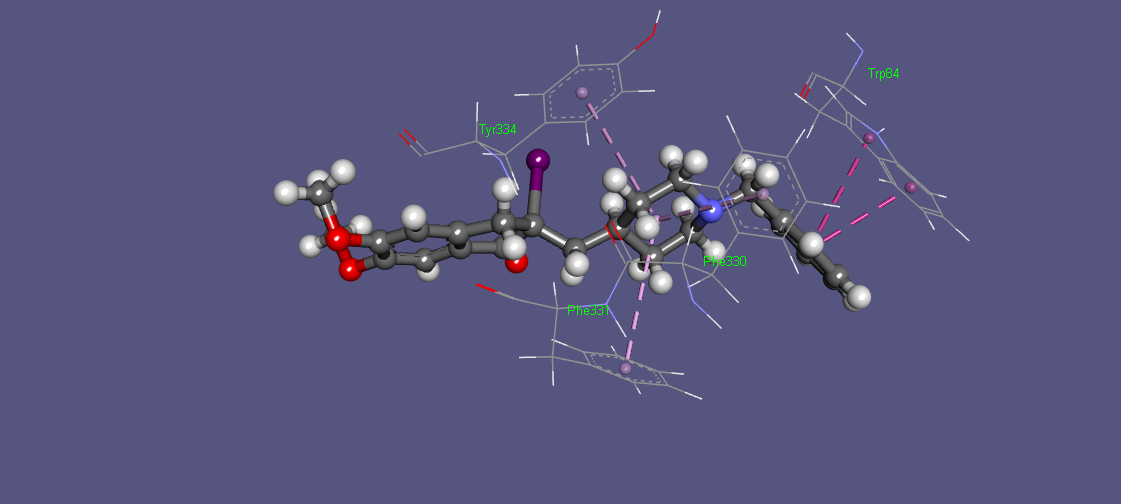
**

(c) **D4**- AChE

**Figure S2**: Non-covalent interaction maps with amino acid residues for (a) **D2 (chair)**-AChE (b) **D3 (Chair)**-AChE and (c) **D4 (Chair)**-AChE complexes

**
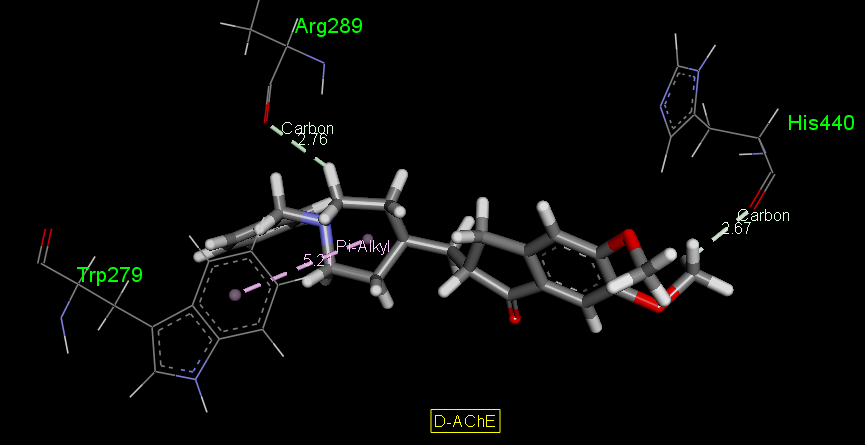
**

(a) **D (boat)** - AChE

**
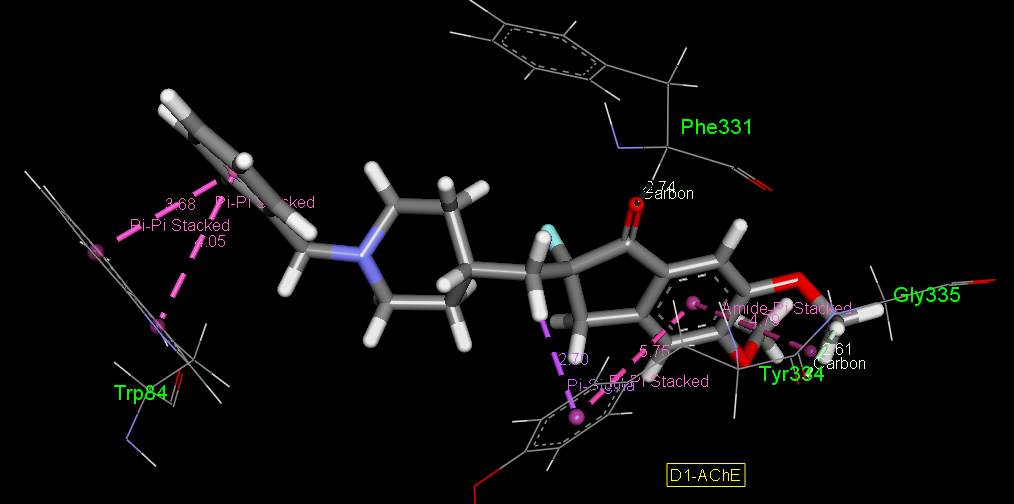
**

(b) **D1 (boat)** - AChE

**
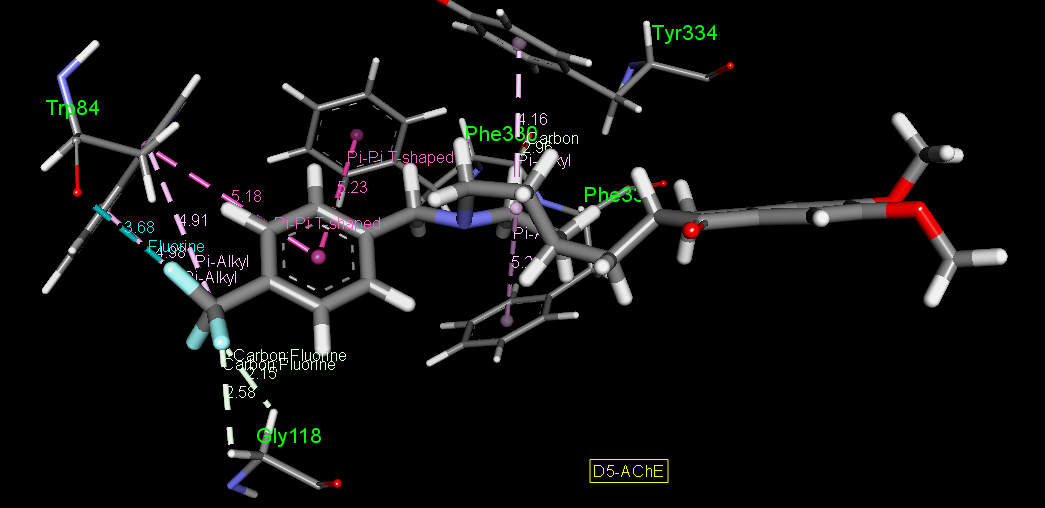
**

(c) **D5 (boat)** - AChE

**Figure S3**: Non-covalent interaction [pi‒pi T shaped, pi‒alkyl, fluorine] maps involving the boat conformers of ligands in (a) **D**-AChE (b) **D1**-AChE and (c) **D5**-AChE complexes

| Name | Stoichiometry | Electronic Energy | Enthalpy | Gibbs Free Energy | Dipole Moment | *S* (Softness) |
| --- | --- | --- | --- | --- | --- | --- |
| **D*** | C_24_H_29_NO_3_ | -1204.876828 | -1204.875884 | -1204.960750 | 3.5713 | 12.160272 |
| **D1*** | C_24_H_28_FNO_3_ | -1303.562601 | -1303.561657 | -1303.647604 | 4.5660 | 13.645357 |
| **D2*** | C_24_H_28_ClNO_3_ | -1662.307882 | -1662.306938 | -1662.394265 | 5.0804 | 15.050041 |
| **D3*** | C_24_H_28_BrNO_3_ | -3766.293888 | -3766.292944 | -3766.381337 | 4.0758 | 13.597117 |
| **D4*** | C_24_H_28_INO_3_ | -8094.417323 | -8094.416379 | -8094.506197 | 4.0041 | 14.114326 |
| **D5*** | C_25_H_28_F_3_NO_3_ | -1540.029054 | -1540.02811 | -1540.121798 | 5.8657 | 11.795234 |

**Table S1.** Stoichiometry, electronic energy, enthalpy, Gibbs free energy (in Hartree), dipole moment (Debye) and softmess of the boat conformers of donepezil and its derivatives.

**Table S2**: Free energy of binding values (Kcalmol^-1^) for ligand – AChE (at chair form) systems obtained from rigid docking

| Systems | Free Energy of Binding |
| --- | --- |
| **D** – AchE | -11.2 |
| **D1** – AchE | -11.6 |
| **D2** – AchE | -11.8 |
| **D3** – AchE | -11.6 |
| **D4** – AchE | -11.2 |
| **D5 –** AchE | -11.5 |
